# Moth Sex Pheromone Receptors and Deceitful Parapheromones

**DOI:** 10.1371/journal.pone.0041653

**Published:** 2012-07-20

**Authors:** Pingxi Xu, Stephen F. Garczynski, Elizabeth Atungulu, Zainulabeuddin Syed, Young-Moo Choo, Diogo M. Vidal, Caio H. L. Zitelli, Walter S. Leal

**Affiliations:** 1 Honorary Maeda-Duffey Laboratory, University of California Davis, Davis, California, United States of America; 2 USDA-ARS, Yakima Agricultural Research Laboratory, Wapato, Washington, United States of America; INRA-UPMC, France

## Abstract

The insect's olfactory system is so selective that male moths, for example, can discriminate female-produced sex pheromones from compounds with minimal structural modifications. Yet, there is an exception for this “lock-and-key” tight selectivity. Formate analogs can be used as replacement for less chemically stable, long-chain aldehyde pheromones, because male moths respond physiologically and behaviorally to these parapheromones. However, it remained hitherto unknown how formate analogs interact with aldehyde-sensitive odorant receptors (ORs). Neuronal responses to semiochemicals were investigated with single sensillum recordings. Odorant receptors (ORs) were cloned using degenerate primers, and tested with the *Xenopus* oocyte expression system. Quality, relative quantity, and purity of samples were evaluated by gas chromatography and gas chromatography-mass spectrometry. We identified olfactory receptor neurons (ORNs) housed in trichoid sensilla on the antennae of male navel orangeworm that responded equally to the main constituent of the sex pheromone, (11*Z*,13*Z*)-hexadecadienal (Z11Z13-16Ald), and its formate analog, (9*Z*,11*Z*)-tetradecen-1-yl formate (Z9Z11-14OFor). We cloned an odorant receptor co-receptor (Orco) and aldehyde-sensitive ORs from the navel orangeworm, one of which (AtraOR1) was expressed specifically in male antennae. AtraOR1•AtraOrco-expressing oocytes responded mainly to Z11Z13-16Ald, with moderate sensitivity to another component of the sex pheromone, (11*Z*,13*Z*)-hexadecadien-1-ol. Surprisingly, this receptor was more sensitive to the related formate than to the natural sex pheromone. A pheromone receptor from *Heliothis virescens*, HR13 ( = HvirOR13) showed a similar profile, with stronger responses elicited by a formate analog than to the natural sex pheromone, (11*Z*)-hexadecenal thus suggesting this might be a common feature of moth pheromone receptors.

## Introduction

Insects achieve their prominence through successful reproduction, which in turn relies heavily on an acute olfactory system. Thousands of finely tuned pheromone sensors in the antennae [1]–[3] enable male moths to follow the trail of a pheromone scent remotely released by conspecific females when they overtly advertise their readiness to mate. The acuteness of the insect's olfactory system is clearly manifested in the selective and sensitive detection of sex pheromone by male moths. Although a single molecule of the natural sex pheromone is estimated to be sufficient to activate a neuron in male antennae [4], pheromone analogs with minimal structural modifications may have very little or no activity [5]. One noticeable exception to this “lock-and-key” tight selectivity of the receptor-pheromone system is that formate analogs are “deceitful” to detectors of aldehyde pheromone. These pheromone analogs, also known as parapheromones [6], were discovered when our understanding of pheromone sensory physiology was still at its infancy. Prior to the discovery of (7*Z*,11*Z*)- and (7*Z*,11*E*)-hexadecedien-1-yl acetate [7] as the major constituents of the sex pheromone of the pink bollworm, *Pectinophora gossypiella*, Shorey and collaborators [8], [9] demonstrated that permeation of the air with a male attractant named hexalure, (7*Z*)-hexadecen-1-yl acetate, led to disruption of pheromonal communication between males and females and resulted in a reduced larval infestation. Subsequently, Mitchell and collaborators [10] tested formate compounds along with other pheromones in an attempt to develop a multispecies mating disruption approach. They observed that (9*Z*)-tetradecen-1-yl formate (hereafter referred to as Z9-14OFor) was highly disruptive of pheromonal communication between male and female corn earworms, *Heliothis* (now *Helicoverpa*) *zea* and tobacco budworms, *Heliothis virescens*, although the pheromones of these species were not used in these field tests. Because the chemical structure of Z9-14OFor resembles that of the major pheromone components of these species, namely, (11*Z*)-hexadecenal ( =  Z11-16Ald) [10], it became evident that formate analogs may be used as replacement for chemically less stable aldehyde pheromones. It was later demonstrated that olfactory receptor neurons (ORNs) involved in the detection of Z11-16Ald, the major component of *H. zea* sex pheromone, responds almost equally to the formate analog, Z9-14OFor [11]. Likewise, it has been shown that a formate analog not only stimulated the ORN sensitive to the major sex pheromone component of the carob moth, *Ectomyelois ceratoniae*, but is also behaviorally active [12].

**Figure 1:**
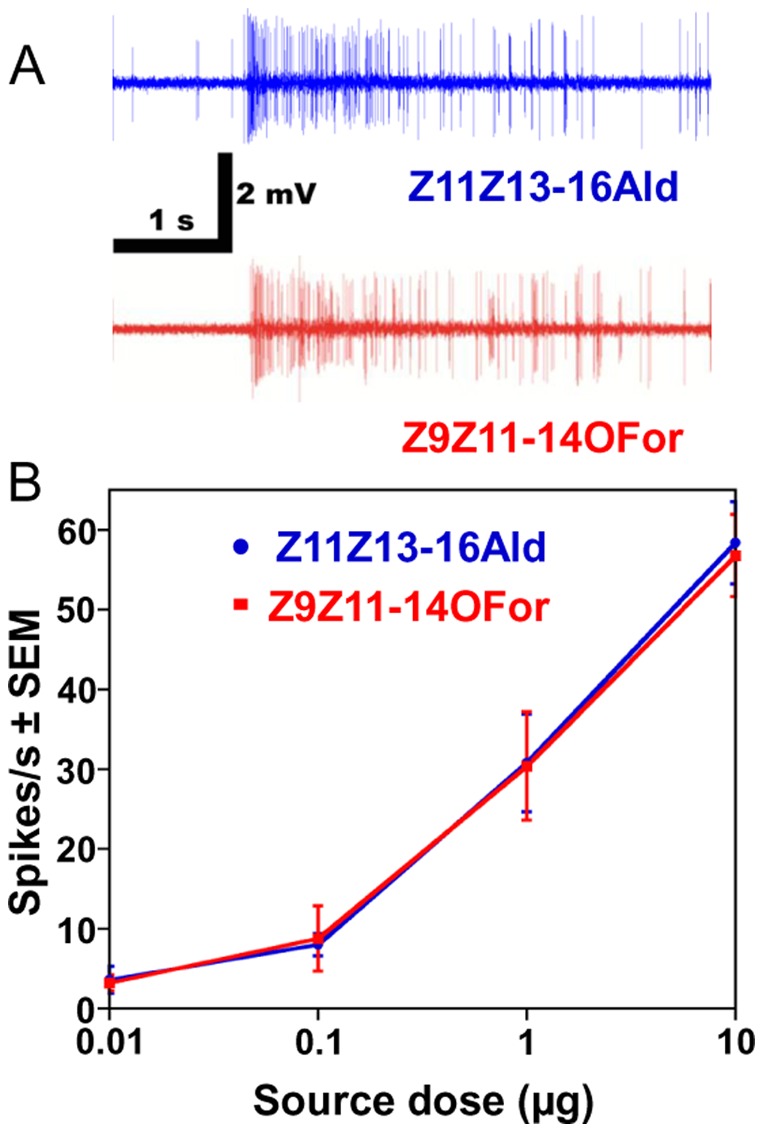
Responses of the peripheral olfactory system of the navel orangeworm to the major constituent of the sex pheromone and its formate analog. (A) Extracellularly recorded single unit responses from ORNs housed in a trichoid sensillum on the antennae, and (B) dose-dependent relationships (*n* = 5; error bar in all figures represent SEM).

**Figure 2:**
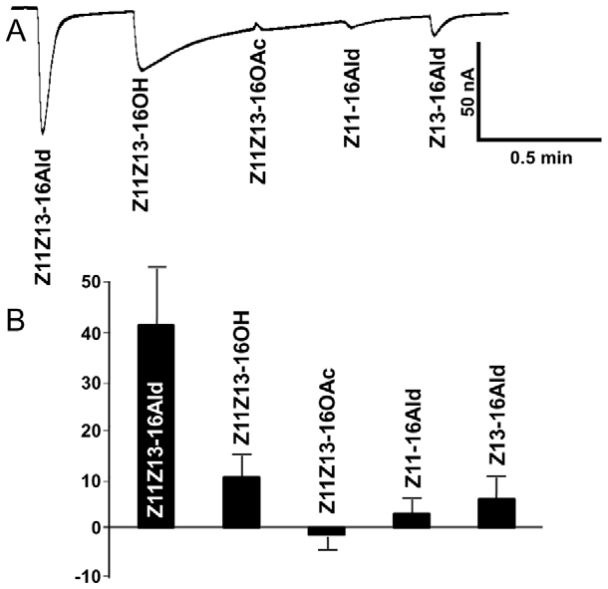
Screening of AtraOR1. *Xenopus* oocytes expressing AtraOR1 and AtraOrco were challenged with three aldehydes released by the female pheromone gland, a related alcohol (Z11Z13-16OH) and a behavioral antagonist (Z11Z13-16OAc). (A) Trace obtained with all compounds at 100 µM. (B) Quantification of current responses (*n* = 5).

**Figure 3:**
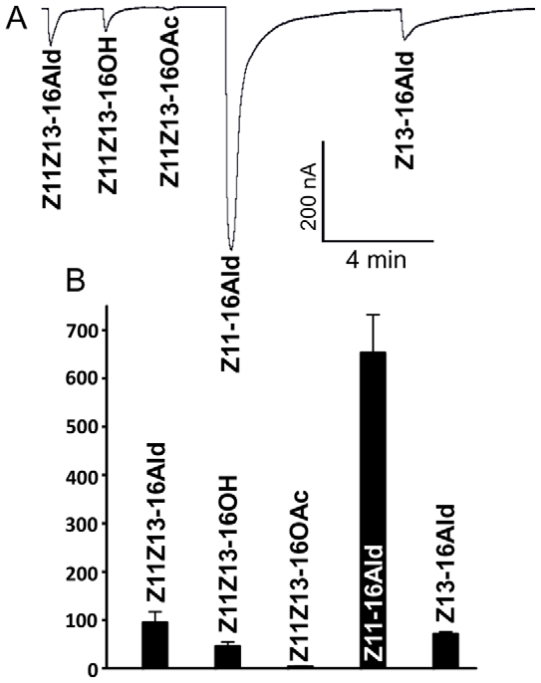
Responses elicited by constituents of the pheromone gland on AtraOR3•AtraOrco-expressing oocytes. (A) Trace generated at 100 µM highlighting a specific response to Z11-16Ald, a gland constituent of unknown function. (B) Quantification of current responses, *n* = 4.

To investigate how this “deceitful” detection of formate analogs is manifested at moth odorant receptor (OR) level, we studied odorant-OR interactions in the navel orangeworm, *Amyelois transitella*, a major pest of the multibillion dollar almond, pistachio and walnut industries. Previously, it has been demonstrated that male-female communication is equally disrupted by the main constituent of the sex pheromone, (11*Z*,13*Z*)-hexadecadienal ( = Z11Z13-16Ald), and its formate analog, (9Z,11Z)-tetradecadien-1-yl formate ( = Z9Z11-14OFor) [13]. With single-sensillum recordings, we first demonstrated that Z11Z13-16Ald and Z9Z11-14OFor elicit indistinguishable responses from ORNs housed in pheromone-detecting trichoid sensilla. Then, we cloned an OR sensitive to the major constituent of the sex pheromone and examined its response profile when expressed in the *Xenopus* oocyte expression system. Surprisingly, the pheromone receptor showed a lower threshold to the formate analog, and the dose-depend curve for the formate was shifted by at least one order of magnitude thus showing a more robust response to the parapheromone (than to the natural sex pheromone). Similar response profiles were observed with a pheromone receptor from *H. virescens* when challenged with its cognate ligand, Z11-16Ald, and its formate analog, Z9-14OFor.

**Figure 4:**
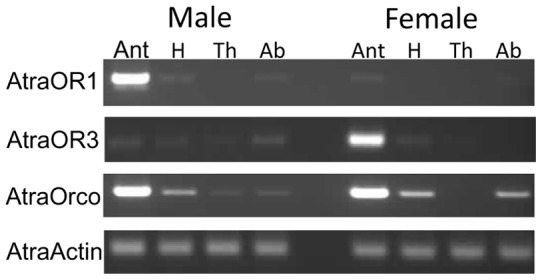
Expression profiles of navel orangeworm odorant receptors. The odorant receptor co-receptor, AtraOrco, was expressed equally in male and female antennae. AtraOR1 was highly expressed in male antennae, whereas AtraOR3 was enriched in female antennae.

**Figure 5:**
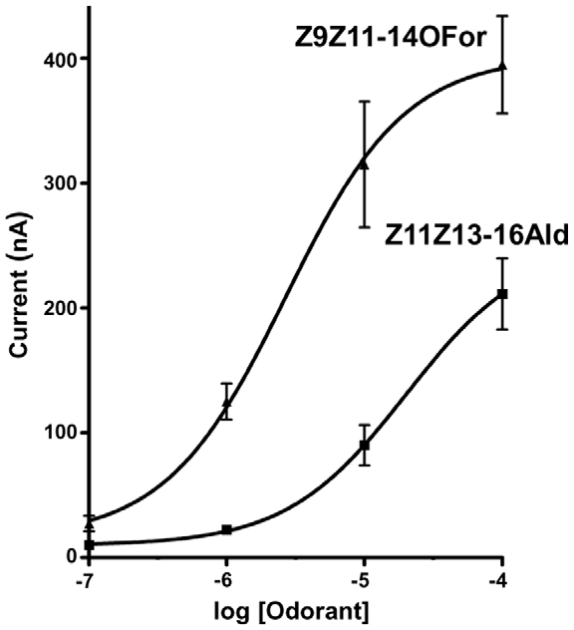
Dose-dependent current responses obtained with AtraOR1•AtraOrco-expressing oocytes. Note the curve generated by challenging the oocytes with the formate analog is shifted at least one order of magnitude.

## Results and Discussion

### Peripheral sensory physiology

The pheromone gland of the navel orangeworm produces a complex semiochemical mixture, which in addition to the main constituent, Z11Z13-16Ald [14], contains a related alcohol, (11*Z*,13*Z*)-hexadecadien-1-ol ( = Z11Z13-16OH), a behavioral antagonist, (11*Z*,13*Z*)-hexadecadien-1-yl acetate ( = Z11Z13-16OAc), highly unsaturated hydrocarbons, monounsaturated aldehydes, Z11-16Ald and Z13-16Ald [15], and other minor constituents [15], [16], but no formates are produced. Behavioral observations in wind tunnel experiments led to the conclusion that a mixture of Z11Z13-16Ald, Z11Z13-16OH, and (3*Z*,6*Z*,9*Z*,12*Z*,15*Z*)-tricosapentaene is necessary and sufficient for full attraction of male moths [17].

**Figure 6:**
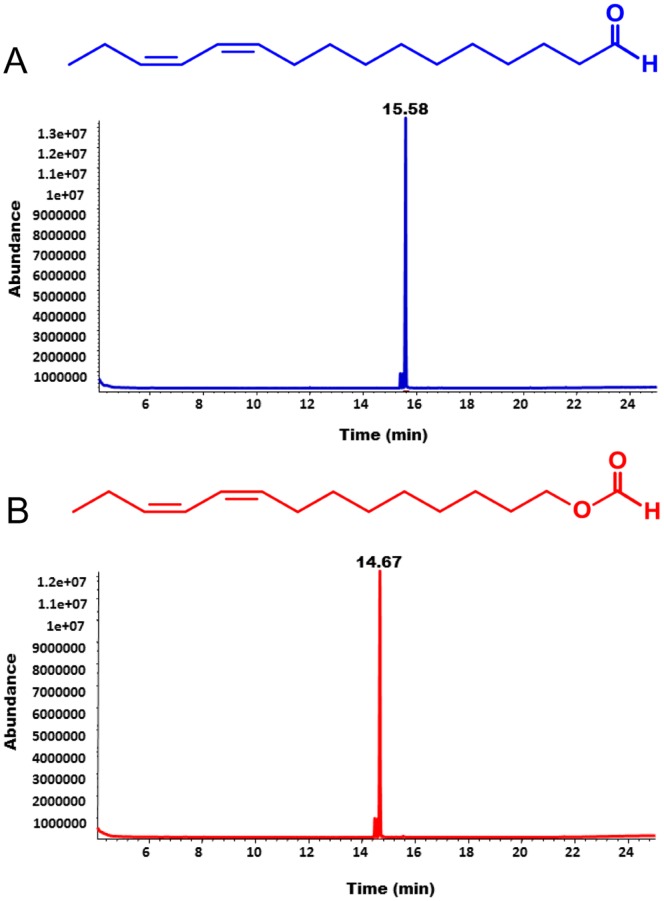
Total ion-chromatograms. Representative traces obtained with the main constituent of the sex pheromone (blue trace) and its related formate (red). Small peaks eluting just prior to the main peaks are stereoisomers of sex pheromone and parapheromone.

**Figure 7:**
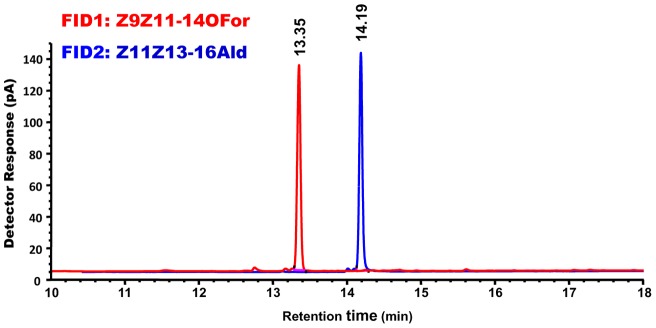
Gas chromatographic traces obtained with extracts of the odorants used to stimulate receptor-expressing oocytes. The peaks of the formate analog and the related aldehyde pheromone are of nearly the same intensity thus confirming that oocytes are stimulated with nearly the same concentrations of the two compounds.

**Figure 8:**
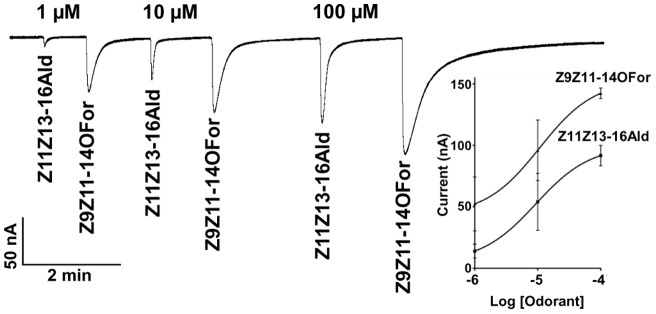
Current responses elicited from AtraOR1•AtraOrco-expressing oocytes by challenging the same oocytes with the aldehyde pheromone and its parapheromone. To avoid electrophysiological adaptation, dose-dependence relationships were obtained with a narrow range of concentration, *n* = 4.

With single sensillum recordings (SSR), we identified at least two distinct populations of long trichoid sensilla that housed ORNs responding to components of the navel orangeworm sex pheromone. One population housed three ORNs, which we named ORN-A, ORN-B, and ORN-C on the basis of their decreasing order of spike amplitudes. While ORN-A responded to Z11Z13-16Ald, ORN-B was activated by another essential constituent of the sex pheromone system, Z11Z13-16OH [17]. ORN-B responded also to ethyl-(*Z*,*Z*)-11,13-hexadecadienoate, a minor component of the sex pheromone gland [15] with unknown function. The neuron with the smallest spike amplitude, ORN-C responded to the behavioral antagonist, Z11Z13-16OAc [15]. The other population of trichoid sensilla predominantly displayed only one ORN, which responded exclusively to Z11Z13-16Ald, and displayed functional properties (spike amplitude, spontaneous frequency and response dynamics) similar to those of ORN-A. Thus, two of the major constituents, the diene aldehyde and alcohol, are detected by long trichoid sensilla.

**Figure 9:**
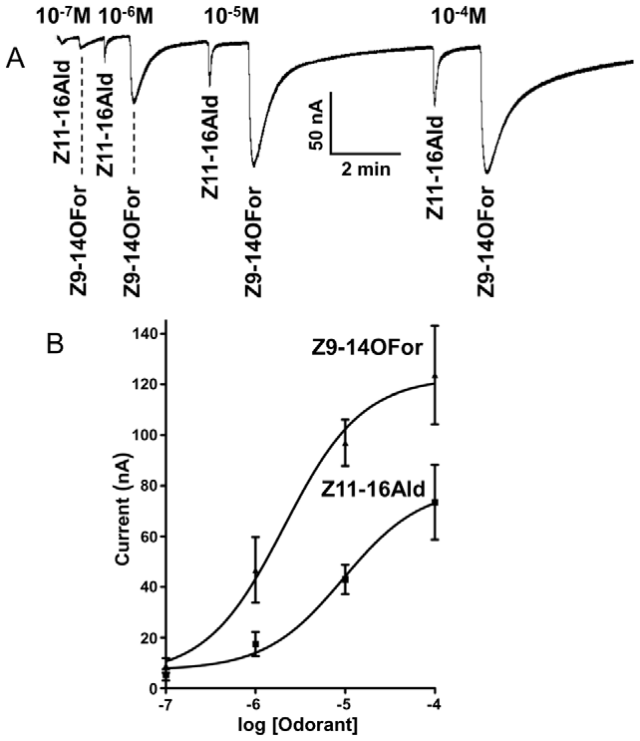
Current responses obtained from HvirOR13•HvirOrco-expressing oocytes challenged with the cognate aldehyde ligand and a related formate analog. (A) Trace generated with increasing doses of the two ligands, and (B) dose-dependent relationships, *n* = 5.

The neurons sensitive to Z11Z13-16Ald were also activated by the formate analog, Z9Z11-14For, with these compounds generating indistinguishable dose-dependent curves (Figure 1). Although not surprising in view of previous observations with other species [11], [12], it is interesting that the olfactory system of male moths is so selective towards pheromones [5] yet it is “deceived” by a pheromone analog. These findings prompted us to investigate whether this “relaxed selectivity” was entirely manifested at the level of an odorant receptor (OR) sensitive to the main constituent of the sex pheromone.

**Table 1:** Oligonucleotide primers used in this study.

Primer Designation	Primer Sequence (5′→3′)	Primer Application
AtraOrcoRACE Fwd	CGGCATGACGCTGCGAGGGGCTGGAGG	AtraOrco 3′ RACE
AtraOrcoRACE Rev	CCCGCCCATGGCATCGAAGGGG	AtraOrco 5′ RACE
AtraOrcoORF Fwd	ATGATAAACAACAAAGTAAAA	AtraOrco ORF cloning
AtraOrcoORF Rev	GTGTTGGTACAACTGAAGTAG	AtraOrco ORF cloning
AtraOrcopGEM Fwd	ATATTCCCGGGATGATAAACAACAAAGTAAAA	AtraOrco pGEMHE cloning
AtraOrcopGEM Rev	AATATTCTAGACTACTTCAGTTGTACCAACAC	AtraOrco pGEMHE cloning
AtraOR1RACE Rev	CCCGCGTACTCTGCGTTGTTACCACTGCTTGCCC	AtraOR1 5′ RACE
AtraOR1ORF Fwd	ATGGATTTTCTATTTGACGCT	AtraOR1 ORF cloning
AtraOR1ORF Rev	TTAACCTTCATTAGTGAATGT	AtraOR1 ORF cloning
AtraOR1pGEM Fwd	ATTATGGATCCATGGATTTTCTATTTGACGCT	AtraOR1 pGEMHE cloning
AtraOR1pGEM Rev	AATAATTCTAGATTAACCTTCATTAGTGAATGT	AtraOR1 pGEMHE cloning
AtraOR1EXP Fwd	TCCGCAAAAATTTCGGATAC	AtraOR1 tissue expression
AtraOR1EXP Rev	CACTTCCACCATCCCCATAG	AtraOR1 tissue expression
AtraOrcoEXP Fwd	AGATGTTGGCTCGTTCTGCT	AtraOrco tissue expression
AtraOrcoEXP Rev	AAGCCGCTTCCATTACTGAC	AtraOrco tissue expression
AtraActinEXP Fwd	GGTCGCGATCTCACAGACTA	AtraActin tissue expression
AtraActinEXP Rev	TCGAGTTGTAGGTGGTTTCG	AtraActin tissue expression
AtraOR3RACE Rev	GATTATTCCTTCAGTGCCATCGTC	AtraOR3 5′ RACE
AtraOR3ORF Fwd	ATGGCTGTATTCACTGAAAGC	AtraOR3 ORF cloning
AtraOR3ORF Rev	TTATTCCTTCAGTGCCATCGT	AtraOR3 ORF cloning
AtraOR3pGEM Fwd	ATATTCCCGGGATGGCTGTATTCACTGAAAGC	AtraOR3 pGEMHE cloning
AtraOR3pGEM Rev	TAATATCTAGATTATTCCTTCAGTGCCATCGT	AtraOR3 pGEMHE cloning
AtraOR3EXP Fwd	CCCGCTAACTTTGATGGTGT	AtraOR3 tissue expression
AtraOR3EXP Rev	GCCACACCCATATCCGTAAC	AtraOR3 tissue expression
HvirOrcopGEMFwd	CCCGGGATGATGACCAAAGTGAAGGCC	HvirOrco pGEMHE cloning
HvirOrcopGEMRev	TCTAGATTACTTGAGTTGTACCAACAC	HvirOrco pGEMHE cloning
HvirOR13pGEMFwd	CCCGGGATGAAAATCCTATCGGACGGT	HvirOR13 pGEMHE cloning
HvirOR13pGEMRev	TCTAGATTATTCTTCTTCTGCAACTGT	HvirOR13 pGEMHE cloning

### Identification of an odorant receptor co-receptor and odorant receptors

Previously, we have identified a partial cDNA sequence from the navel orangeworm encoding a putative OR83-like odorant receptor [18], which we renamed AtraOrco given the new nomenclature for odorant receptor co-receptors [19]. We have now obtained the full-length sequence of AtraOrco (1425 bp, 475 amino acid residues; GenBank accession number JX173647). With degenerate primers designed on the basis of known moth OR sequences [20] we cloned two putative OR, which we named AtraOR1 (1284 bp, 428 amino acid residues; GenBank accession number JX173648) and AtraOR3 (1272 bp, 424 amino acid residues; GenBank accession number JX173649) with 43-47% identity to aldehyde-sensitive moth pheromone receptors from *H. virescens* [21], [22], *Diaphania indica*, *Plutella xylostella* [23], and *Antheraea pernyi* [24]. We named the newly identified ORs in order of discovery and skipped OR2 so to avoid possible confusion with AtraOrco.

Next, we tested the response profiles of these ORs when co-expressed with AtraOrco in the *Xenopus* oocyte expression system. Although both ORs are sensitive to aldehyde constituents of the sex pheromone, AtraOR1•AtraOrco was activated by Z11Z13-16Ald (Figure 2) with almost no response to monounsaturated aldehydes previously identified in the pheromone gland [15]. In contrast, AtraOR3•AtraOrco responded mainly to Z11-16Ald (Figure 3), with relatively low response to Z11Z13-16Ald. We noticed with interest that the AtraOR1•AtraOrco-expressing oocytes showed moderate response to Z11Z13-16OH and minor, but “inverse,” response to the behavioral antagonist, Z11Z13-16OAc (Figure 2), which is a semiochemical produced by the navel orangeworm that behaviorally affects allospecific male moths [15]. Since Z11-16Ald is not an essential constituent of the sex pheromone [17] and consequently there is no evidence that its related formate is behaviorally active, we decided to select AtraOR1 to investigate whether a pheromone receptor is equally activated by a known aldehyde pheromone [14] and its behaviorally active formate analog [13].

We then performed RT-PCR analysis to determine whether our selected OR is indeed expressed in male antennae as implied by the above-described electrophysiological recordings. Not surprisingly, AtraOrco was expressed in both male and female antennae, with minor expression in other non-olfactory tissues (Figure 4), while expression of AtraOR3 was biased to female antennae. By contrast, RT-PCR demonstrated that AtraOR1 is expressed specifically in male antennae thus suggesting its putative role in pheromone reception.

### AtraOR1 is “tricked” by a formate analog

Surprisingly, AtraOR1•AtraOrco-expressing oocytes responded more strongly to the synthetic formate analog, Z9Z11-14OFor, than to the natural sex pheromone component Z11Z13-16Ald (Figure 5). The formate analog showed not only a lower threshold, but the dose-dependence curve for the parapheromone was shifted by at least one order of magnitude, with EC_50_ of 499 nM and 90 nM for aldehyde and formate, respectively.

We are cognizant that vapor pressure differences may account in part for the indistinguishable dose-dependent responses observed by SSR (Figure 1). As the boiling point of formate analogs are lower than the those of related aldehydes, puffs at the same source dose are expected to release relatively more molecules of a formate than an aldehyde of equivalent molecular weight (e.g.: Z9Z11-14OFor vs. Z11Z13-16Ald). However, in the *Xenopus* oocyte recording system vapor pressure differences are not relevant as odorants are delivered in aqueous phase. To make certain that AtraOR1•AtraOrco-expressing oocytes were subjected to nearly equal concentrations of the two ligands, we carefully prepared dilutions of the two odorants, extracted aliquots with organic solvent and analyzed the extracts by gas chromatography-mass spectrometry (GC-MS) (Figure 6). Identity of the major peak in each sample was confirmed by its mass spectrum (data not shown). As expected for high purity samples and given the inert nature of the DMSO containing Ringer solution, the nominal and measured concentrations of the two odorants were nearly identical (Figure 7). To further avoid possible variations of individual oocytes and/or electrophysiological adaptation of oocytes, we challenged the same oocyte preparations with both odorants in increased order of response and with a limited range of concentrations (1–100 µM). Clearly, the response to identical doses of Z9Z11-14OFor elicited higher responses than those triggered by Z11Z13-16Ald (Figure 8). These findings demonstrate that in the *Xenopus* oocyte recording system an OR sensitive to an aldehyde pheromone was not only deceived by a formate analog, but also responded to the parapheromone with higher sensitivity than to the natural constituent of the sex pheromone system.

### Response of *H. virescens* pheromone receptor to a formate analog

To compare our findings using a pheromone-sensitive OR from the navel orangeworm with a known pheromone receptor from another moth species, we tested an aldehyde receptor from *H. virescens*, HR13 ( = HvirOR13) when expressed with its co-receptor, HR2 ( = HvirOrco). With clones kindly provided by Dr. Jurgen Krieger (University of Hohenheim, Germany), we prepared HvirOR13•HvirOrco-expressing oocytes. We prepared samples of the main constituent of the sex pheromone, Z11-16Ald, and a behaviorally active formate analog, Z9-14OFor, analyzed aliquots to make certain they were of the same concentration, and then challenged oocyte preparations. As observed with the oocytes expressing the navel orangeworm OR, HvirOR13•HvirOrco-expressing oocytes were activated more strongly by the formate analog than the natural sex pheromone (Figure 9), as indicated by the EC_50_ (150 nM for Z11-16Ald; literature, 367 nM in [21] and 33 nM for Z9-14OFor). It is therefore likely that stronger response elicited by a formate analog than to the natural aldehyde pheromone is a common feature of moth ORs in line with practical observations that these esters can be used to replace less stable aldehydes in mating disruption [25], [26] and other pheromone applications.

## Materials and Methods

### Electrophysiology

Single sensillum (unit) recordings (SSR) were performed as previously reported [27]. *Xenopus laevis* oocytes were purchase from EcoCyte Bioscience (Austin, TX). Chemical-induced currents were recorded with the two-electrode voltage-clamp technique at holding potential of −80mV. Signals were amplified with an OC-725C amplifier (Warner Instruments, Hamden, CT), low-pass filtered at 50 Hz and digitized at 1 kHz. Data acquisition and analysis were carried out with Digidata 1440A and software pCLAMP 10 (Molecular Devices, LLC, Sunnyvale, CA).

### Chemical and chemical analysis

(11*Z,*13*Z*)-hexadecadienal and (9*Z,*11*Z*)-tetradecadien-1-yl formate were gifts from Bedoukian Research Inc. (Dubnary, CT). (11*Z*)-hexadecenal and (9*Z*)-tetradecen-1-yl formate were purchased from Plant Research International (Wageningen, The Netherlands). For SSR, samples were diluted with glass-distilled hexane to make 10 µg/µl stock solutions from which decadic dilutions were made. For *Xenopus* oocyte recordings, stock solutions were prepared in dimethyl sulfoxide (DMSO) and stored at −20°C until use. Just prior to use they were brought to room temperature and while an oocyte preparation was being washed with Ringer's solution, stock solutions were diluted in 0.1% DMSO-containing 1x Oocyte Ringer's solution [NaCl 96 mM, KCl 2 mM, CaCl_2_ 1.8 mM, MgCl_2_ 1 mM, HEPES 5 mM, pH 7.6]. The concentrations of ligands in figures are nominal (undiluted) concentrations, which were used to challenge the oocyte preparations. First, OR-expressing oocytes were screened with 100 µM of ligands, which is a typical dose used for screening [28]. Dose-dependent curves were obtained with lower concentrations; buffer alone (control) generate no detectable inward curent. EC_50_s were calculated on the basis of the actual concentrations the oocytes were exposed to, i.e., the concentration after dilution with the buffer bathing oocyte preparations. Individual aliquots (200 µl) of same aldehyde and formate samples that were used to stimulate oocytes were extracted with hexane (200 µl) and analyzed by gas chromatography-mass spectrometry (GC-MS). Their concentrations were compared by quantifying these hexane extracts by gas chromatography (GC). These chemical analyses were performed as previously described [29], with a different temperature program. The oven of the GC-MS was operated at 70°C (100°C for GC), held at this initial temperature for 1 min, increased at 10°C/min to 290°C (250°C for GC), and held at this final temperature for 10 min (5 min for GC).

### Cloning AtraOR1, AtraOrco, and AtraOR3

To obtain the nucleotide sequence of a full-length cDNA encoding AtraOrco, a 5′ and 3′ rapid amplification of cDNA ends (RACE) strategy was utilized. RACE primers were designed from a partial cDNA sequence previously identified [18]. Total RNA was extracted from antennae dissected from navel orangeworm males with TRIzol reagent (Invitrogen, Carlsbad, CA) according to manufacturer's protocol and the resultant RNA was treated with DnaseI (Biolabs, New England) to remove genomic DNA contaminants. For 5‵ and 3‵RACE, the SMARTer^TM^ RACE cDNA amplification kit (Clontech, Mountain View, CA) was used according to manufacturer's protocol. Briefly, first strand cDNA was synthesized from 1μg of total RNA at 42°C for 90 min using SMARTScribe^TM^ Reverse Transcriptase with either the 5‵ and 3‵-CDS primer and the SMARTer II A oligonucleotide (Clontech). RACE PCRs were performed with the Advantage GC polymerase kit (Clontech) using sequence specific primers, AtraOrcoRACE Fwd or AtraOrcoRACE Rev (Table 1) and universal primer mix according to the manufacture's protocol (Clontech).

Touchdown PCR with the appropriate RACE cDNA and primers was performed under the following amplification program: 94°C, 30 s to activate Advantage GC polymerase followed by 5 cycles of two segment PCR at 94°C, 30 s and 72°C, 3 min, then 5 cycles of three segment PCR at 94°C, 30 s; 70°C, 30 s and 72°C, 3 min and 40 cycles of 94°C, 30 s; 68°C, 30 s and 72°C for 3 min. Final extension was performed at 72°C for 6 min, RACE-PCR fragments were cloned into PBluescript and analyzed by sequencing. The complete cDNA sequence of the ORF encoding AtraOrco was obtained by PCR using gene specific primers, AtraOrcoORF Fwd and AtraOrcoORF Rev (Table 1) and the above RACE cDNA. PCR was performed using Advantage GC polymerase and the following cycling parameters: initial denaturation at 94°C for 30 s; followed by 40 cycles of 94°C, 30 s; 68°C, 30 s and 72°C for 3 min; and a final extension at 72°C for 6 min. PCR fragments were cloned into pBluescript and sequenced. To clone the ORF encoding AtraOrco into pGEMHE, primers with restriction endonuclease sites were designed AtraOrcopGEM Fwd and AtraOrcopGEM Rev (Table 1) and used in PCR reactions with conditions as above and resultant PCR products were cloned into pGEMHE [30]. The nucleotide sequence obtained from the pGEMHE clones were identical to those determined for AtraOrco cloned into pBluescript.

Initial amplification of AtraOR1 was done using a degenerate primer/3′RACE approach as previously described [20]. Briefly, the 3′ end of the cDNA sequences encoding potential pheromone receptors were amplified in PCR reactions using the forward primers PR0 and PR6 [20] and the reverse primer, CDSIII 3′PCR (Clontech, Mountain View, CA). PCR products were amplified from male antennal cDNA in 20 µl reactions with Titanium® Taq (Clontech, Mountain View, CA). PCR products were visualized on 1.2% agarose gels, excised, and cloned using the TOPO TA cloning kit for sequencing (Invitrogen, Carlsbad, CA) with TOP 10 *E. coli* chemically competent cells. Plasmid DNA was extracted from picked colonies using the QIAprep spin mini prep kit (Qiagen, Valencia, CA) and the cDNA clones were sequenced at MC Laboratories (MCLab, San Francisco, CA). A sequence specific primer, AtraOR1RACE Rev (Table 1) for use in 5′RACE was designed from the cDNA sequence information obtained from the degenerate primer amplifications above. To amplify the 5′ nucleotide sequence encoding AtraOr1, 5′ RACE reactions were performed as above using AtraOR1RACE Rev and universal primer mix (Clontech). To amplify the complete ORF of AtraOR1, primers AtraOR1ORF Fwd and AtraOR1ORF Rev (Table 1) were used in PCR reactions using the following conditions: 94°C, 30 s to activate Advantage GC polymerase, followed by 40 cycles of 94°C, 30 s; 68°C, 30 s and 72°C for 3 min, with final extension performed at 72°C for 6 min. The resultant products were cloned into pPCR-Script and the nucleotide sequence was determined. A cDNA containing the ORF encoding AtraOR1 was cloned into pGEMHE by PCR using the sequence specific primers, AtraOR1pGEM Fwd and AtraOR1pGEM Rev with added restriction endonuclease sites (Table 1). PCR conditions were as mentioned above.

Initial amplification of AtraOR3 was done as described above for AtraOR1. A partial gene transcript of 913 nt encoding for 61 amino acids was amplified using the degenerate primer 3′RACE procedure. To obtain the 5′ nucleotide sequence of the gene transcript encoding AtraOR3, 5′RACE reactions were done using the SMART™ RACE cDNA Amplification Kit with a sequence specific primer, AtraOR3RACE Rev (Table 1). After sequencing, the cDNA containing the sequence encoding for AtraOR3 was cloned into pGEMHE by PCR with AtraOR3pGEMHE Fwd and AtraOR3pGEMHE Rev specific primers, which contain restriction endonuclease sites (Table 1).

### Analysis of receptor expression in navel orangeworm

Tissues were collected from adult male and female antennae, heads, thoraces, and abdomen and placed in 100 µl RNAlater® (Ambion, Austin, TX). Total RNA was extracted using the RNeasy® Plus Mini Kit (Qiagen, Valencia, CA) according to the manufacturer's protocol, and quantitated using the Quant-iT™ RiboGreen® RNA assay kit (Invitrogen, Carlsbad, CA). For each tissue type, 100 ng of total RNA was converted to cDNA with SuperScript® III First-Strand Synthesis SuperMix (Invitrogen, Carlsbad, CA) using manufacturer's supplied oligo d(T)_20_ and protocol. PCR amplifications were done using sequence specific primers (200 nM final concentration) to detect AtraOR1 (AtraOR1EXP Fwd and AtraOR1EXP Rev; Table 1), AtraOrco (AtraOrcoEXP Fwd and AtraOrcoEXP Rev; Table 1) and Actin (AtraActinEXP Fwd and AtraActinEXP Rev; Table 1). PCR products were amplified from cDNA template equivalent to 5 ng of input RNA in 25 µl reactions with Titanium® Taq (Clontech) and the following conditions: initial denaturation for 3 min at 94°C; then amplification for 20 s at 94°C; 20 s at 62°C; 30 s at 72°C for 35 cycles; followed by a final 5 min 72°C incubation. PCR products were separated by loading 1/4^th^ of the total reaction onto 1.5% agarose gels and visualized on a UV light box. To confirm identity of PCR products, bands were excised, TA cloned and sequenced as above.

### *In vitro* transcription and oocyte microinjection

Capped RNA (*in vitro* transcription) was prepared using a mMESSAGE mMACHINE T7 Kit (Ambion) according to the manufacturer's protocol. Templates plasmids were fully linearized with Nhe I, and capped cRNA was transcribed using T7 RNA polymerase. Purified cRNAs were re-suspended in nuclease-free water at a concentration of 200 µg/µl and stored at −80°C. RNA concentration was determined by UV spectrophotometry (Smartspec 3000, Bio-Rad). *Xenopus laevis* oocytes on stage V or VI were microinjected with 2 ng of an OR and 2 ng of an Orco. Injected oocytes were incubated at 18°C for 3–7 days in modified Barth's solution [NaCl 88 mM, KCl 1 mM, NaHCO_3_ 2.4 mM, MgSO_4_ 0.82 mM, Ca(NO_3_)_2_ 0.33 mM, CaCl_2_ 0.41 mM, HEPES 10 mM, pH 7.4] supplemented with 10 µg/ml of gentamycin, 10 µg/ml of streptomycin and 1.8 mM sodium pyruvate.

### Cloning of HvirOrco and HvirOR13 into pGEMHE

Clones of HvirOrco ( = HR2, Accession No. AJ487477) and HvirOR13 ( = HR13, AJ748328) were gifts from Dr. Jurgen Krieger (University of Hohenheim, Germany). To clone their ORFs into pGEMHE, the following primers with restriction endonuclease sites were designed: HvirOrcopGEM Fwd and HvirOrcopGEM Rev and HvirOR13pGEM Fwd and HvirOR13pGEM Rev (Table 1), respectively. PCR amplifications were performed with PfuUltraTM II Fusion HS DNA polymerase (Agilent Technologies, Santa Clara, CA) under the following condition: 94°C for 5 min, 33 cycles of 94°C for 30 s, 55°C for 40 s, 72°C for 2 min, and 72°C for 10 min. PCR products were cloned into PCR-Script Amp Cloning vector (Agilent Technologies, Santa Clara, CA) before being cloned into pGEMHE. Plasmids were extracted using the QIAprep spin mini prep kit (Qiagen, Valencia, CA) and sequenced using ABI 3730 automated DNA sequencer at Davis Sequencing (Davis, CA).
